# Oxidative stress genes in patients with esophageal squamous cell carcinoma: construction of a novel prognostic signature and characterization of tumor microenvironment infiltration

**DOI:** 10.1186/s12859-022-04956-9

**Published:** 2022-09-30

**Authors:** Wei Liu, Hao-Shuai Yang, Shao-Yi Zheng, Hong-He Luo, Yan-Fen Feng, Yi-Yan Lei

**Affiliations:** 1 Department of Thoracic Surgery, The First Affiliated Hospital, Sun Yat-Sen University, Guangzhou, 510080 Guangdong China; 2 State Key Laboratory of Oncology in South China, Collaborative Innovation Center for Cancer Medicine, Sun Yat-Sen University Cancer Center, Guangzhou, 510060 Guangdong China; 3 Department of Pathology, Sun Yat-Sen University Cancer Center, Guangzhou, 510060 Guangdong China

**Keywords:** Esophageal squamous cell carcinoma, Oxidative stress, Prognosis, Immune infiltrates, Marker

## Abstract

**Background:**

Oxidative stress plays an important role in the progression of various types of tumors. However, its role in esophageal squamous cell carcinoma (ESCC) has seldom been explored. This study aimed to discover prognostic markers associated with oxidative stress in ESCC to improve the prediction of prognosis and help in the selection of effective immunotherapy for patients.

**Results:**

A consensus cluster was constructed using 14 prognostic differentially expressed oxidative stress-related genes (DEOSGs) that were remarkably related to the prognosis of patients with ESCC. The infiltration levels of neutrophils, plasma cells, and activated mast cells, along with immune score, stromal score, and estimated score, were higher in cluster 1 than in cluster 2. A prognostic signature based on 10 prognostic DEOSGs was devised that could evaluate the prognosis of patients with ESCC. Calculated risk score proved to be an independent clinical prognostic factor in the training, testing, and entire sets. P53 signaling pathway was highly enriched in the high-risk group. The calculated risk score was positively related to the infiltration levels of resting mast cells, memory B cells, and activated natural killer (NK) cells and negatively associated with the infiltration levels of M1 and M2 macrophages. The relationship between clinical characteristics and risk score has not been certified. The half-maximal inhibitory concentration (IC50) values for sorafenib and gefitinib were lower for patients in the low-risk group.

**Conclusion:**

Our prognostic signature based on 10 prognostic DEOSGs could predict the disease outcomes of patients with ESCC and had strong clinical value. Our study improves the understanding of oxidative stress in tumor immune microenvironment (TIME) and provides insights for developing improved and efficient immunotherapy strategies.

**Supplementary Information:**

The online version contains supplementary material available at 10.1186/s12859-022-04956-9.

## Background

Esophageal cancer (EC) is a highly aggressive malignancy with poor prognosis, ranking sixth and seventh highest in mortality and morbidity worldwide, respectively. Two main histopathological variants of EC have been described, including esophageal adenocarcinoma and esophageal squamous cell carcinoma (ESCC), which differ significantly in incidence, etiology, and clinical characteristics [1, 2]. Chinese patients account for 70% of all the EC patients worldwide [3]. ESCC is the most common pathological subtype of EC in China, accounting for approximately 90% of all the cases [1]. Most patients are diagnosed at an advanced stage, and such patients show poor response to surgery, poor outcome, and high recurrence rate [4].

Accumulating evidence over the recent years suggest, that oxidative stress plays a key role in the pathogenesis of EC [5–7]. Oxidative stress refers to a state of imbalance between oxidation and antioxidation in the body; which leads to inflammatory infiltration of neutrophils [8]. An increasing number of studies have found that oxidative stress and its consequent damage are important factors involved in the development of cancers [9–11], such as breast cancer [12, 13], ovarian cancer [14, 15], lung cancer [16, 17], liver cancer [18, 19], and EC [20, 21]. Oxidative stress promotes cell carcinogenesis via dysregulation of oxidation and antioxidation, and by generation and elimination of reactive oxygen species [22, 23]. Accumulating evidence suggests the potential of implications of modulating oxidative stress-related processes in cancer therapy [24–26]. Oxidative stress has been reported to modulate immune cell activity in ovarian cancer, and is related to the immune microenvironment [27]. Similarly, a certain relationship between oxidative stress-induced apoptosis and immune microenvironment has also been observed in patients with gastric and esophageal cancers, which can influence the prognosis of patients [28]. To the best of our knowledge, very few studies have explored the relationship between ESCC and oxidative stress, and the pathophysiology still remains unknown. In addition, the relationship between oxidative stress and immune microenvironment in ESCC requires further investigation.

In this study, clinical information and RNA-seq expression profiles corresponding to patients with ESCC were retrieved from gene expression omnibus (GEO) and the cancer genome atlas (TCGA) database. We performed consensus cluster analysis on the basis of expression of differential oxidative stress genes between ESCC and normal tissues. To improve the predictive performance of the differential oxidative stress gene signature, we constructed a risk model using a training set and verified this model using a testing set. We also evaluated the predictive value and diagnostic efficacy of this model and determined tumor immune infiltration and medical treatment using it in patients with ESCC.

## Results

### Differential expression of genes and functional enrichment analysis

**Fig. 1:**
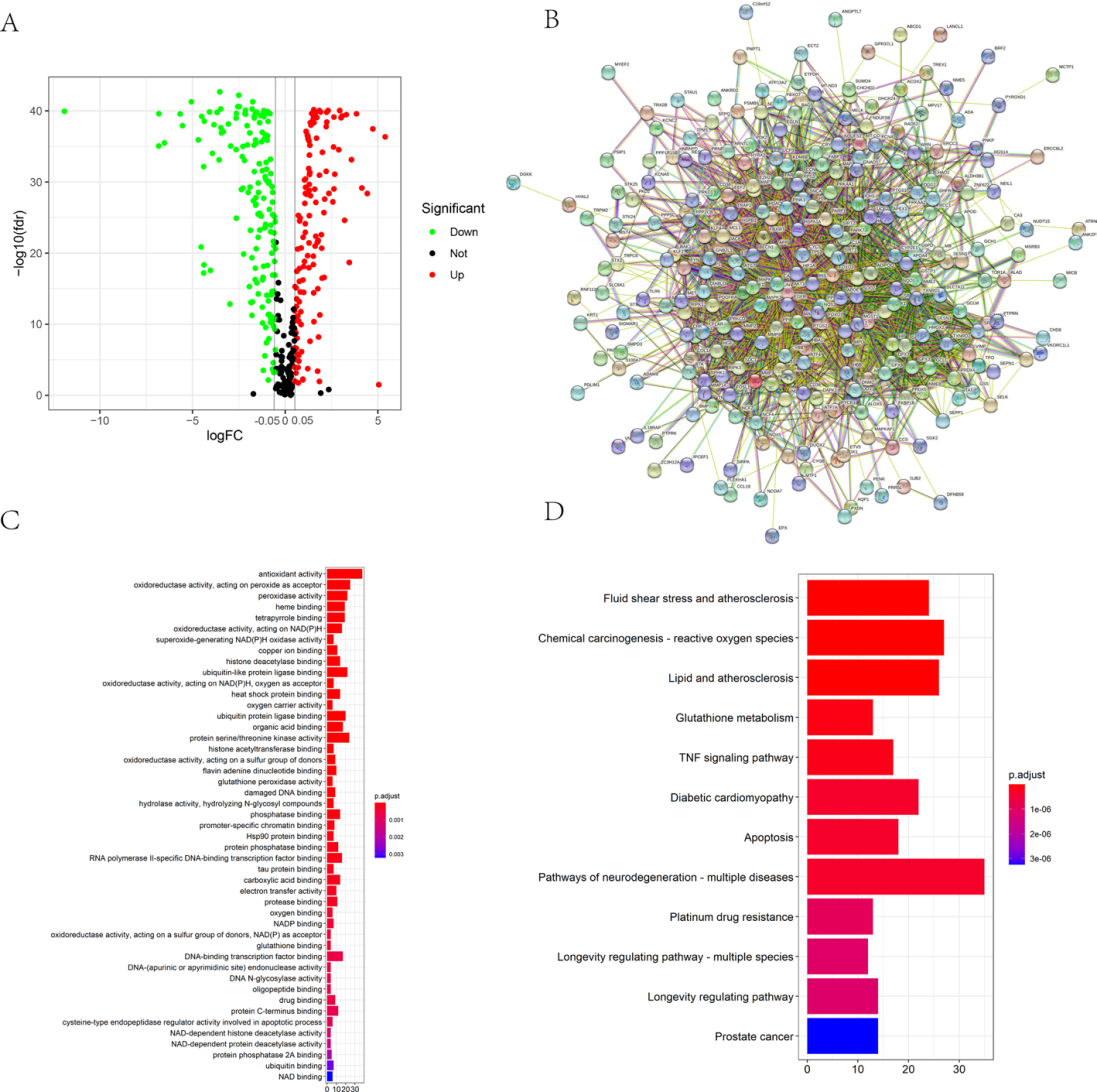
**A** Volcano map of DEOSGs between normal and ESCC samples. **B** Network between DEOSGs. **C**, **D** Bubble plots of GO analyses (**C**) and KEGG analyses (**D**)

Oxidative stress gene matrices were obtained from 650 normal samples (GTEx) and 77 ESCC samples (TCGA) for differential analysis of gene expression. Consequently, we obtained 294 differentially expressed oxidative stress-related genes (DEOSGs), visualized as volcano plot (Fig. 1A). Of these 294 DEOSGs, 133 were upregulated, while the remaining were downregulated. We utilized the STRING database to observe the interactions among these 294 DEOSGs, and after deleting the genes without interactions, a protein–protein interaction network of DEOSGs was constructed (Fig. 1B). Subsequently, these 294 DEOSGs were subjected to functional enrichment analysis designed to elucidate their biological processes and pathways activities. Gene ontology (GO) analysis revealed that antioxidant activity, peroxide, NADPH, heat shock protein, serine threonine kinase, and ubiquitinated protein ligase were significantly enriched (Fig. 1C), whereas Kyoto encyclopedia of genes and genomes (KEGG) analysis showed that chemical carcinogens, tumor necrosis factor (TNF) signaling pathways, apoptosis, and platinum resistance were significantly enriched (Fig. 1D).

### Identification of two clusters of patients with ESCC

**Fig. 2:**
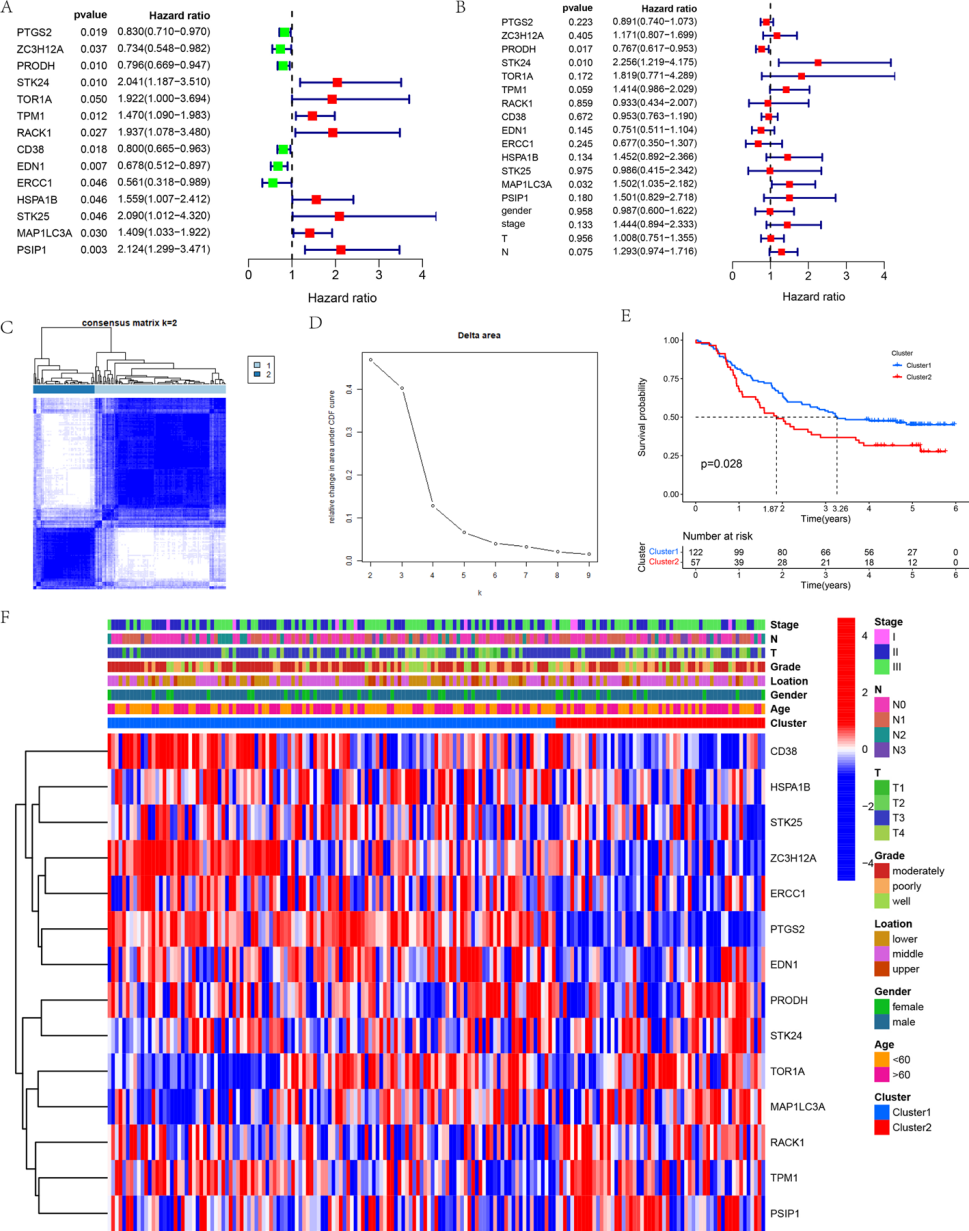
**A** The prognostic DEOSGs extracted by Univariate Cox regression analysis. **B** The independent prognostic DEOSGs extracted by Multivariate Cox regression analysis. **C**, **D** Unsupervised clustering of 14 prognostic DEOSGs in the GEO-ESCC cohort: **C** Relative change in area under CDF curve for k = 2 to 9; **D** The ESCC cohort from GEO was divided into two distinct clusters when k = 2. **E** Kaplan–Meier survival curve of patients between cluster 1 and 2. **F** Comparison of the relationship between the clinical characteristics of two clusters and heatmap of 14 prognostic DEOSGs. Blue represents down-regulation and red represents up-regulation of genes

Univariate Cox proportional hazards regression analysis showed that 14 DEOSGs were significantly related to overall survival (OS) (*p* < 0.05). PTGS2, ZC3H12A, PRODH, CD38, EDN1, and ERCC1 were protective factors with hazard ratios (HRs) of < 1 and STK24, TOR1A, TPM1, RACK1, HSPA1B, STK25, MAP1LC3A, and PSIP1 were risk factors with HRs of > 1 (Fig. 2A). Three independent prognostic DEOSGs (PRODH, STK24, and MAP1LC3A) were identified by multivariate Cox proportional hazards regression analysis after consideration of confounding factors such as gender, tumor, node, and metastasis (TNM) stage, tumor (T) stage, and node (N) stage (Fig. 2B). We then constructed a consensus cluster based on 14 prognostic DEOSGs expressions. Figure 2C shows the change curve of cumulative distribution function (CDF) from k = 2 to k = 9 for the consensus cluster; k = 2 was the best value when the area under the curve (AUC) was the largest, so we divided the patient population with ESCC derived from the GEO database into two clusters (Fig. 2D). Kaplan–Meier analysis showed that cluster 1 had a significant survival advantage (*p* = 0.028, Fig. 2E). Moreover, the median survival time of ESCC patients was higher in cluster 1 than in cluster 2 (3.26 vs. 1.87 years). Figure 2F shows comparison of clinical characteristics and expression levels of 14 prognostic DEOSGs between the two clusters. Significant differences were not found between the two clusters 1 and 2 in terms of sex, age, location, TNM stage, T stage, or N stage. The expression levels of CD38, HSPA1B, STK25, ZC3H12A, ERCC1, PTGS2, EDN1, and PRODH were lower in cluster 2 than in cluster 1, and the expression levels of STK24, TOR1A, MAP1LC3A, RACK1, TPM1, and PSIP were lower in cluster 1 than in cluster 2.

### Estimation of immune cell infiltration in the cluster subgroups

**Fig. 3:**
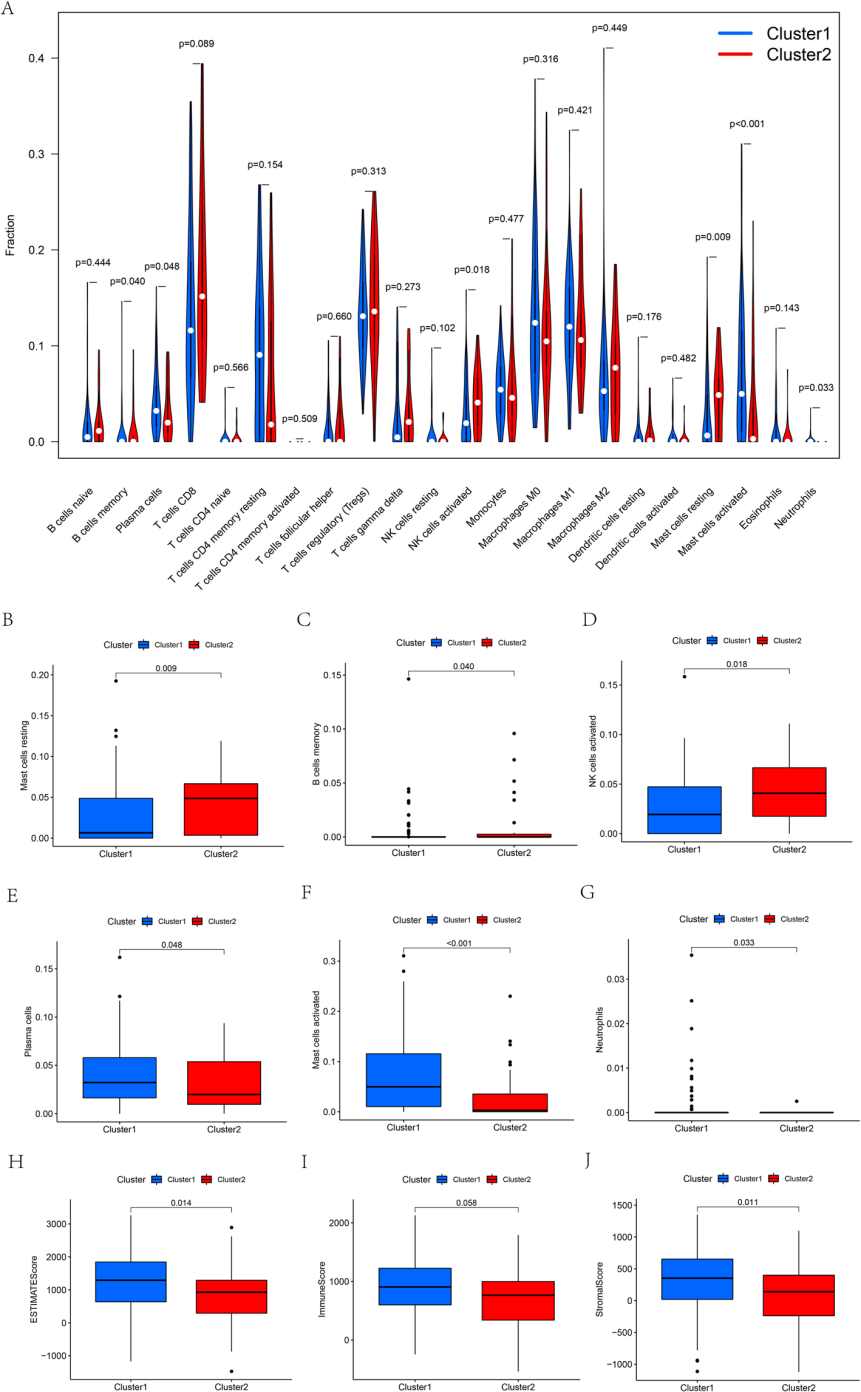
**A**–**G** The infiltrating levels of 22 immune cell types in cluster1 vs cluster 2: **B** resting mast cells, **C** memory B cells, **D** NK cells, **E** plasma cells, **F** activated mast cells, **G** neutrophils. The comparison of immune-related scores between cluster 1 and cluster 2 (**H**, **J**): (**H**) estimated score, **I** immune score, **J** stromal score

We found that cluster 2 exhibited higher infiltration levels of memory B cells, resting mast cells, and natural killer (NK) cells than cluster 1 (Fig. 3A–D). The infiltration levels of neutrophils, plasma cells, and activated mast cells were lower in cluster 2 than in cluster 1 (Fig. 3E–G). In addition, we calculated tumor immune microenvironment (TIME) scores and found that cluster 2 showed comparatively lower levels of immune score, estimated score, and stromal score (*p* < 0.05, Fig. 3H–J).

### Establishment and verification of risk assessment model

**Fig. 4:**
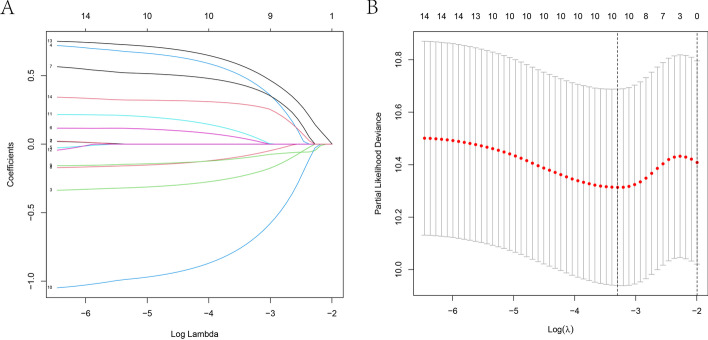
**A**, **B** The prognostic signature constructed by the minimum criterion of LASSO Cox regression algorithm

We performed Lasso regression on 14 prognostic DEOSGs to properly fit the prognostic signature, and obtained 10 DEOSGs (PRODH, STK24, TPM1, RACK1, CD38, EDN1, ERCC1, HSPA1B, MAP1LC3A, and PSIP1); wherein the first-order probability of deviation of log (λ) was minimal (Fig. 4A, B). The formula used to calculate the risk score for each patient with ESCC was as follows:

$$\begin{aligned} {\text{Risk}}\;{\text{score}} &= {\text{PRODH}} \times \left( { - 0.{\text{2153}}0{\text{727}}0{\text{28836}}} \right) + {\text{STK24}} \times \left( {0.{\text{4557632981}}0{\text{3475}}} \right) \\ & \quad + {\text{TPM1}} \times \left( {0.0{\text{2926121}}0{\text{8251768}}} \right) + {\text{RACK1}} \times \left( {0.{\text{41}}0{\text{189196857841}}} \right) \\ & \quad + {\text{CD38}} \times \left( { - 0.0{\text{75994}}0{\text{749876345}}} \right) + {\text{EDN1}} \times \left( {0.0{\text{9313397838}}0{\text{6189}}} \right) \\ & \quad + {\text{ERCC1}} \times \left( {0.{\text{7}}00{\text{516244351}}0{\text{63}}} \right) + {\text{HSPA1B}} \times \left( {0.0{\text{58}}0{\text{781289297423}}} \right) \\ & \quad + {\text{MAP1LC3A}} \times \left( {0.{\text{5394642534}}0{\text{6876}}} \right) + {\text{PSIP1}} \times \left( {0.{\text{281871899388918}}} \right). \\ \end{aligned}$$

**Fig. 5:**
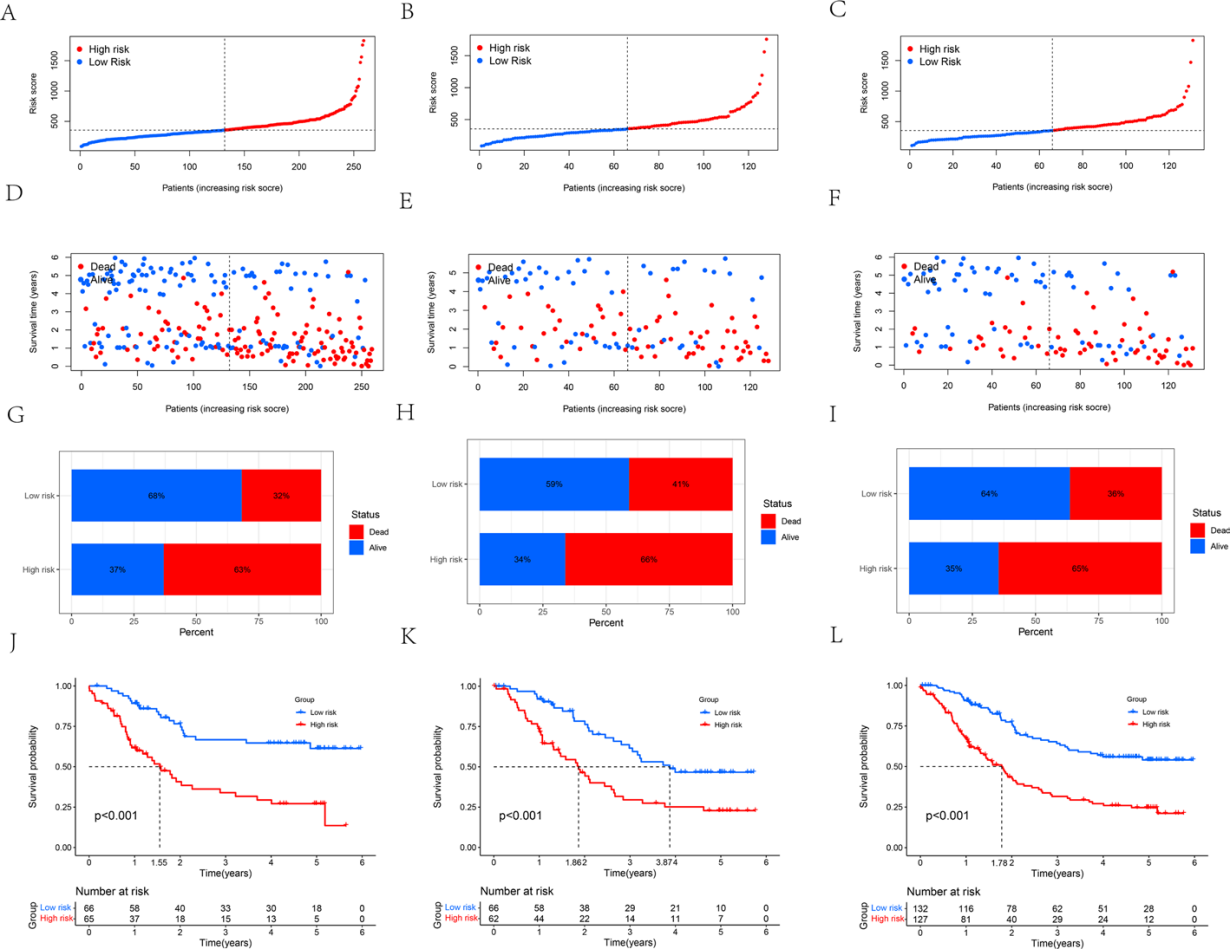
**A**–**C** Risk score distribution of patients between high- and low-risk groups in the training (**A**), testing (**B**), and entire sets (**C**), respectively. **D**–**I** Survival status of patients between high- and low-risk groups in the training (**D**, **G**), testing (**E**, **H**), and entire sets (**F**, **I**), respectively. **J**–**L** Kaplan–Meier survival curve of patients between high- and low-risk groups in the training (**J**), testing (**K**), and entire set (**L**), respectively

**Fig. 6:**
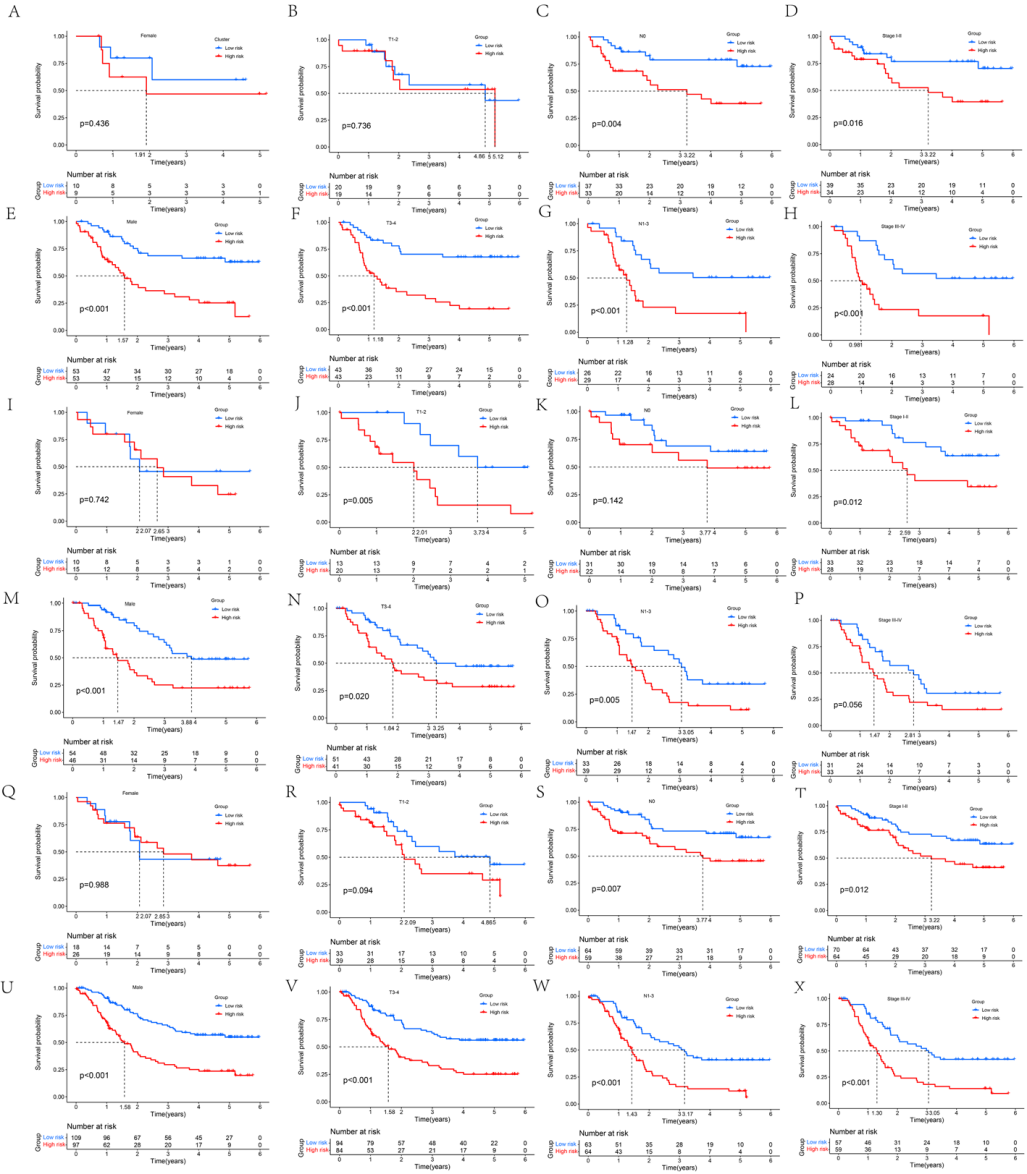
**A**–**H** Kaplan–Meier survival curves stratified by gender (**A**, **E**), stage T (**B**, **F**), N (**C**, **G**), or TNM (**D**, **H**) between low- and high-risk groups in the training set. **I**–**P** Kaplan–Meier survival curves stratified by gender (**I**, **M**), stage T (**J**, **N**), N (**K**, **O**), or TNM (L, P) between low- and high-risk groups in the testing set. **Q**–**X** Kaplan–Meier survival curves stratified by gender (**Q**, **U**), stage T (**R**, **V**), N (**S**, **W**), or TNM (**T**, **X**) between low- and high-risk groups in the entire set

The specific risk score and risk level group for each patient with ESCC is shown in Additional file 1: Table S1. The distribution of risk score, survival status, and survival time between low- and high-risk group patients was compared. The results obtained from training, testing, and entire sets showed that the high-risk group had poorer prognosis than the low-risk group (Fig. 5A–L). Median survival time of patients with ESCC in the high-risk group in the training set was 1.55 years, while the median survival time was not reached in the low-risk group (*p* < 0.001). Median survival time of patients with ESCC was significantly higher in the low-risk group than in the high-risk group in the testing set (3.87 vs 1.86 years; *p* < 0.001). Median survival time of patients with ESCC in the high-risk group in the entire set was 1.78 years, while the median survival time was not reached in the low-risk group (*p* < 0.001). Moreover, with the exceptions of female ESCC in the training, testing and entire sets, T1-2 ESCC in training and entire sets, N0 ESCC in the test set and stage III-IV ESCC in the test set, the results of male, stage I-IV, T stage, and N stage in the training, testing, and entire sets also showed that low-risk group had better prognoses (Fig. 6A–X). Median survival times off the ESCC high- and low- risk group patients, and their survival curves stratified by these clinical characteristics in the training, testing, and entire sets are shown in Additional file 2: Table S2.

Additional file 3: Fig. S1A, B, D, E, G, and H show the comparison of expression levels of 10 prognostic DEOSGs between the low- and high-risk groups. Expression levels of PRODH, CD38, and ERCC1 in the training, testing, and entire sets were notably lower in the high-risk group than in the low-risk group. Expression levels of MAP1LC3A and PSIP1 in the training, testing, and entire sets were higher in the high-risk group than in the low-risk group.

We observed the following positive correlations between the expressions of DEOSGs in the training, testing, and entire sets: EDN1 with ERCC1 and PRODH; PSIP1 with RACK1 and TPM1; and STK24 with PRODH. We also observed that the following negative correlations between the expressions of DEOSGs: CD38 with RACK1 and MAP1LC3A; CD38 with RACK1 and MAP1LC3A; and PSIP1 and TPM1 with PRODH (Additional file 3: Fig. S1C, F, and I).

### Evaluation of the risk model

**Fig. 7:**
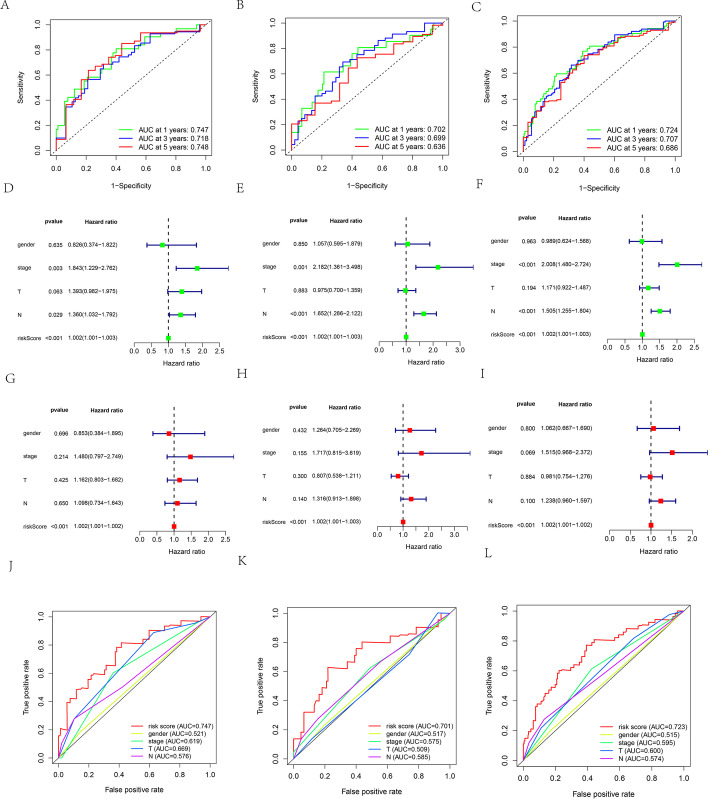
**A**–**C** Time-dependent ROC curve analyses of risk score in the training (**A**), testing (**B**), and entire sets (**C**), respectively. **D**–**F** Univariate Cox analyses of clinical factors and risk score with OS in the training (**D**), testing (**E**), and entire sets (**F**), respectively. (**G**–**I**) Multivariate Cox analyses of clinical factors and risk score with OS in the training (**G**), testing (**H**), and entire sets (**I**), respectively. (**J**–**L**) One-year ROC curve analyses of gender, clinical stage, T stage, N stage and risk score in the training (**J**), testing (**K**), and entire sets (**L**), respectively

We used time-dependent receiver operating characteristic (ROC) curves to assess the specificity and sensitivity of the risk model and found that the 1-, 3-, and 5-year AUCs were as follows: 0.75, 0.72, and 0.75 for the training set (Fig. 7A); 0.70, 0.70, and 0.64 for the testing set (Fig. 7B); and 0.72, 0.71, and 0.69 for the entire test (Fig. 7C), respectively. The results of univariate and multivariate Cox proportional hazards regression analyses between clinical features and risk score in the training, testing, and entire sets are shown in Additional files 4, 5: Tables S3 and 4. In the training, testing, and entire sets (Figs. 7D, E, and F), univariate Cox regression analysis showed that TNM stage (*p* = 0.003, HR = 1.843, 95% confidence interval (CI) [1.229–2.762]; *p* = 0.001, HR = 2.182, 95%CI [1.361–3.498]; *p* < 0.001, HR = 2.008, 95%CI [1.480–2.724], respectively), N stage (*p* = 0.029, HR = 1.360, 95% CI [1.032–1.792]; *p* < 0.001, HR = 1.652, 95%CI [1.286–2.122]; *p* < 0.001, HR = 1.505, 95%CI [1.255–1.804], respectively), and risk score (*p* < 0.001, HR = 1.002, 95%CI [1.001–1.003]; *p* < 0.001, HR = 1.002, 95%CI [1.001–1.003]; *p* < 0.001, HR = 1.002, 95%CI [1.001–1.003], respectively) showed significant differences (Fig. 7D, E, and F), whereas multivariate Cox regression analysis showed that risk score (*p* < 0.001, HR = 1.002, 95%CI [1.001–1.002]; *p* < 0.001, HR = 1.002, 95%CI [1.001–1.003]; *p* < 0.001, HR = 1.002, 95%CI [1.001–1.003], respectively) was an independent prognostic predictor (Figs. 7G, H, and I). We found the AUC values for gender, stage, T, and N in one-year survival to be as follows: training set, 0.521, 0.619, 0.669, and 0.576, (Fig. 7J); testing set, 0.517, 0.575, 0.509 and 0.585, (Fig. 7K); entire set, 0.515, 0.595, 0.600 and 0.574, respectively (Fig. 7L).

The strip charts (Additional file 6: Fig. S2A, F, and K) and consequent scatter diagrams showed that gender, clinical stage, T stage, and N stage were not associated with the risk score in the training (Additional file 6: Fig. S2B, C, D, and E, respectively), testing (Additional file 6: Fig. S2G, H, I, and J, respectively), and entire sets (Additional file 6: Fig. S2L, M, N, and O, respectively).

### Comparison of gene set variation analysis between low- and high-risk groups

**Fig. 8:**
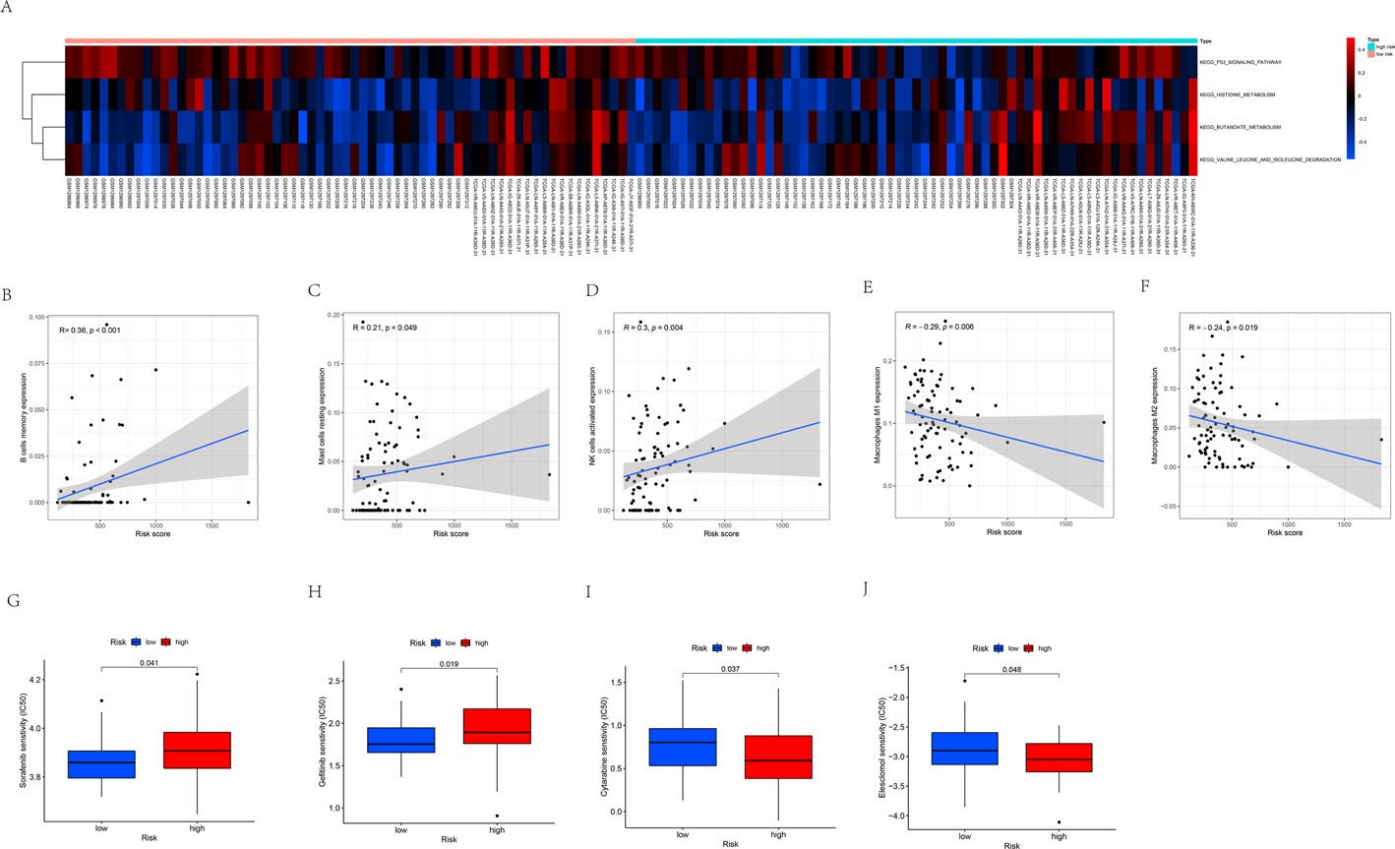
**A** GSVA enrichment analysis between high- and low-risk groups in the training. The heatmap was used to visualize these biological processes, and red represented activated pathways and blue represented inhibited pathways. **B**–**F** The correlation between risk score and the infiltration levels of immune cells: (**B**) memory B cells, **C** resting mast cells, **D** activated NK cells, **E** macrophages M1, **F** macrophages M2. **G**–**J** The sensitivity to drugs in high- and low-risk score patients: (**G**) Sorafenib, **H** Gefitinib, **I** Cytarabine, **J** Elesclomol

We then compared the differences in the biological behaviors between high-risk and low-risk groups in the training set using the KEGGs pathway enrichment analysis. P53 signaling pathway was highly enriched in the high-risk group, and the enrichment level was closely related to tumor aggressiveness. On the contrary, histidine metabolic pathway, methyl butyrate metabolic pathway, and valine leucine isoleucine degradation pathway were more enriched in the low-risk group than in the high-risk group (Fig. 8A).

### Assessment of clinical treatment and immunity factors in the risk model

A positive association was observed between risk score and infiltration levels of memory B cells (*p* < 0.001, Fig. 8B), resting mast cells (*p* = 0.049, Fig. 8C), and activated NK cells (*p* = 0.004, Fig. 8D). In addition, risk score was found to be negatively related to the infiltration of M1 (*p* = 0.006, Fig. 8E) and M2 (*p* = 0.019, Fig. 8F) macrophage enrichment. Patients in the low-risk group had a lower IC50 for sorafenib and gefitinib than the high-risk group (Figs. 8G-H). Patients in the low-risk group had higher IC50 values for cytarabine and elesclomol than the high-risk group (Fig. 8I–J).

## Discussion

ESCC is a progressive disease, and the effects of existing treatments for this cancer type are far from satisfactory due to its high recurrence and metastasis rates [29, 30]. Recent studies have focused on construction of gene-based signatures to predict the disease outcomes of patients with ESCC [31, 32]. Several studies have explored the therapeutic potential of regulating oxidative stress in cancer [33, 34], however, very few studies have focused on ESCC. Therefore, it is crucial to further explore this topic.

In this study, we explored the expression patterns and prognostic values of oxidative stress signatures that constituted a model and TIME in the context of ESCC. In our study, the expression levels of PTGS2, CD38, STK25, ZC3H12A, EDN1, TOR1A, and MAP1LC3A in normal tissues were lower than the ESCC tissues. We found that high expression levels of PTGS2, CD38, and EDN1 were associated with better prognosis in patients with ESCC, and this finding was in concordance with previous research [35–37]. Elevated expression levels of MAP1LC3A have been claimed to be related to poor outcome in patients with ESCC [38]. In this study, the expression levels of HSPA1B, ERCC1, PRODH, STK24, RACK1, TPM1, and PSIP were found to be notably higher in normal tissues than in ESCC tissues. Low expression levels of ERCC1 have been reported to be related to improved prognosis in patients with ESCC [39]. Increased expression levels of RACK1 and TPM1 have been claimed to be related to poor outcome in patients with ESCC [40, 41]. Other genes included in this study have not been previously studied in patients with ESCC, but they have been claimed to be independent prognostic factors in patients with cancer. The following genes and their expression levels were claimed to be related to poor outcome in patients with different types of cancers: hepatocellular carcinoma with high STK25 expression levels [42]; colorectal cancer with low ZC3H12A expression levels [43]; colon cancer with elevated HSPA1B expression levels [44]; lung adenocarcinoma with STK24 expression levels [45]. PRODH expression has been reported to be related to apoptosis in patients with breast cancer [46]. Our study showed that ZC3H12A, ERCC1, PTGS2, CD38, EDN1, and PRODH expressions were independent factors predicting good prognosis in patients with ESCC. In addition, we also found that STK24, STK25, TOR1A, MAP1LC3A, RACK1, HSPA1B, TPM1, and PSIP expressions were independent factors predicting poor outcome in patients with ESCC, and this finding was consistent with other reports. Then, we constructed a consensus cluster based on the expression of 14 prognostic DEOSGs. Based on DEOSG expressions and specific HRs in the two clusters, we could hypothesize that the prognosis of cluster 1 was better than cluster 2 of patients with ESCC.

Oxidative stress processes have been reported to affect the TIME of patients and influence the prognosis of patients with EC [28]. Mast cell-derived TNF activates the endothelial cells within blood vessels. Neutrophils circulating in the blood are directly activated to migrate into the inflamed tissues. KEGG analysis of 294 DEOSGs revealed the enrichment of TNF pathway, and we theorized that oxidative stress genes may play an important role in mast cell and neutrophil immunoregulatory processes [47]. We further compared the TIME of the two clusters. The infiltration levels of plasma cells, activated mast cells, and neutrophils were higher in cluster 1 than in cluster 2. Moreover, cluster 1 exhibited high levels of the estimated score, stromal score, and immune score. The infiltration levels of memory B cells, resting mast cells, and NK cells in cluster 1 were lower than cluster 2. In our study, cluster 1 was classified as immune-infiltrated type with adaptive immune cell infiltration, stromal, and immune activation. On the other hand, cluster 2 can be classified as immune-inflamed type, characterized by immunodepletion and immune response diminution, which may be the reason for worse prognosis for ESCC patients in cluster 2 [48, 49].

We further established a ten-gene prognostic signature composed of PRODH, CD38, EDN1, ERCC1, STK24, TPM1, RACK1, MAP1LC3A, PSIP1 and HSPA1B using the training set of 131 patients. We found that the calculated risk score could better predict the outcome of patients with ESCC. Moreover, the potential of oxidative stress prognostic signatures was verified using the testing and entire sets, which revealed a great prognostic potential of this risk model.

We verified that the expression of CD38 was negatively related to RACK1 and MAP1LC3A expressions. Low expression of RACK1 and high expression of CD38 were found to be independent factors predicting good prognosis, and high expression of MAP1LC3A was related to unfavorable prognosis in patients with ESCC. These findings were in accordance with previous studies [36, 38, 40]. Our study further illustrates that CD38, RACK1, and MAP1LC3A may serve as strong prognostic biomarkers for ESCC. High calculated risk score was found to be significantly related to poor prognosis of patients with ESCC and it was verified to be an independent prognostic factor which was not influenced by clinical characteristics including sex, T stage, N stage, and TNM stage.

We further analyzed the GSVA and TIME differences in low- and high-risk groups and explored the reasons for the difference in prognosis between the two groups of ESCC patients. High expression of p53 was related to low OS of patients with ESCC [50, 51]. High histidine levels were related to a reduced risk of developing ESCC [52]. The expression level of valine was higher in patients with metastatic ESCC than in non-metastatic ESCC [53, 54]. CD38 can downregulate metabolic signaling pathways associated with p53 [55, 56], and our study also showed a negative correlation between CD38 expression and risk score, consistent with GSVA enrichment analysis results. ERCC1 participates in p53-related metabolic signaling pathways and is a potential target for cancer therapy [57]. Our study also showed a positive correlation between ERCC1 expression and risk score, which was consistent with GSVA enrichment analysis results. In addition, p53 and ERCC1 are used in conjunction to assess tumor malignancy [58], and the effect of chemotherapy response [59, 60]. Depletion of HSPA1B in tumor cells induced macrophage suppression of cytokine-1 expression, and this was identified as a target of p53 tumor suppressor protein [61]. Our study suggests that 10 DEOSGs were associated with P53 signaling pathway and are potential targets for cancer therapy.

In our study, the calculated risk score on the basis of 10 prognostic DEOSGs was positively associated with infiltration levels of memory B cells, resting mast cells, and activated NK cells; and negatively related to the infiltration levels of M1 and M2 macrophages. Low-risk group could be classified as immune-infiltrated type with immune response at peak and adaptive immune cell infiltration, While the high-risk group could be termed as immune-inflamed type, characterized by immunodepletion and immune response diminution [48, 49]. High mast cell density has been related to low OS in patients with ESCC [62]. In general, M1 macrophages exert antitumor effects. In contrast, M2 macrophages promote tumor growth [63]. But an increasing number of studies have shown that this simple classification does not fully reflect the complexity of macrophage function, as macrophages usually adjust their function according to the tissue microenvironment [64, 65]. The single cell profile of ESCC also hints towards that the coexistence of M1 and M2 macrophages in ESCC suggests a more complex macrophage activation pattern than classical M1/M2 model [49]. This phenomenon has also been reported in breast and liver cancer [66, 67]. Our study further suggested the complexity of macrophage regulation of immune function in ESCC TIME, along with the fact that oxidative stress may be associated with macrophage exerting immunomodulatory effects in ESCC [68, 69]. The 10 screened DEOSGs may help in further investigation of the role of macrophages in ESCC. Prognostic signatures of 10 DEOSGs may play a key role in defining the TIME in patients with ESCC.

Although we assessed our model using several methods, this study still had several limitations. The clinical profile information of ESCC patients obtained from TCGA was less rich compared to that obtained from the GEO; therefore, our clinical profile analysis was limited to sex, TNM stage, T stage, and N stage. Although we had collected all ESCC information from the TCGA and GSE53625 series, the sample size of this study was relatively small. Moreover, we used the testing and entire sets to perform internal validation of the model. However, it would be beneficial to improve the sample size and perform external validation using other clinical datasets in future works.

## Conclusions

The study classified ESCC into two subtypes based on the expression of 14 prognostic DEOSGs and explored the differences in TIME. Moreover, we demonstrated a prognostic signature based on 10 DEOSGs that could predict the disease outcomes of patients with ESCC. Risk score was proved to be an independent clinical prognostic factor. Our study improves the understanding of oxidative stress in the TIME and provides more insight into effective immunotherapy strategies. Further studies are necessary to verify our findings, and future work should include in vitro and in vivo verification.

## Methods

### Data acquisition

In this study, clinical information, such as sex, T stage, N stage, metastasis (M) stage, TNM stage, survival, and RNA-seq expression profiles of patients with ESCC were retrieved from GEO database (179 patients; GSE53625, GPL18109) and the TCGA database (95 patients; https://portal.gdc.cancer.gov). Fifteen patients from the TCGA database were excluded due to lack of survival time data. A total of 259 patients (entire set) with prognostic clinical information were randomly divided into training group (131 patients; half of each of the cohorts obtained from the TCGA and GEO databases) and testing group (128 patients; the remaining half of each cohort obtained from these databases) using R package “caret.” RNA-seq expression profiles of 650 normal samples were retrieved from the genotype-tissue expression (GTEx) database.

### Screening of oxidative stress-related genes

A total of 444 oxidative stress genes were obtained from oxidative stress gene set, M3223.gmt, in the Gene Set Enrichment Analysis (GSEA) (http://www.gsea-msigdb.org/gsea/index.jsp).

### Differential expression and enrichment analysis of genes in EC and normal esophageal tissue

DEOSGs were identified by comparing the tissues of 80 ESCC patients from the TCGA-ESCC dataset and 650 normal tissues samples from the GTEx dataset with a threshold for false discovery rate of < 0.05, along with |log2 FC (fold-change) |> 0.5 using R package “limma”. Interacting genes/proteins (STRING, version 11.5, http://string-db.org/) is an online tool that helps study gene or protein interactions, and facilitates visualization [70]. We utilized R package “clusterProfiler” to perform KEGG and GO enrichment analyses to investigate the biological processes associated with these DEOSGs at a significance of *p* < 0.05.

### Clusters based on DEOSGs

Using the DEOSGs retrieved from TCGA and GEO, we performed univariate Cox proportional hazard regression analysis to select genes associated with survival (*p* < 0.05). We explored the potential molecular bastards of ESCC in GEO based on prognostic DEOSG expressions using R package “ConsensusClusterPlus” [71], and divided the patients into two clusters [72].

### Establishment and validation of the risk signature

Once prognostic DEOSGs were obtained from univariate Cox proportional hazards regression analysis, we performed a tenfold cross-validated Lasso regression with a significance threshold of *p* = 0.05, and ran 1000 cycles in the training risk group. To prevent overfitting, 1000 random stimuli were established for each cycle. A model was then developed using the training test. The 1-, 3-, and 5-year receiver ROC curves of the model were plotted using a computational program. The formula used to calculate the risk score was as follows:

$$risk\;score = \mathop \sum \limits_{i = 1}^{k} \beta iSi$$

The Akaike's information criterion (AIC) value at each point of the 5-year ROC curve was assessed to determine the maximum knee as a cut-off point for classifying patients into high- and low-risk groups according to risk scores.

We employed Kaplan Meier analysis and used survival curves to evaluate the difference in survival between the high- and low-risk groups. The specific risk score value for each sample in the model was also visualized using the “survival,” “survminer,” “survivalROC,” and “glmnet” R packages. We performed univariate and multivariate Cox proportional hazards regression analyses for clinical features and risk score using the “survival” R package to confirm whether the risk score could prove to be an independent clinical prognostic predictor, and to construct a 1-year ROC curve to compare the risk score and clinical features. Wilcoxon signed-rank test was performed to compare the risk score differences between groups with different clinical features. We also verified these in the testing set of 128 patients, and in the entire set of 259 patients.

### Investigation of TIME and immune checkpoints

CIBERSORT (http://cibersort.stanford.edu/) is a powerful deconvolution algorithm based on gene expression that can calculate the infiltration levels of immune cells from the gene expression profiles of complex tissues [73]. Using the expression profile of ESCC retrieved from the GEO, TCGA databases and CIBERSORT software, we computed the infiltration levels of 22 infiltrating immune cells, and analyzed the corresponding differences between the clusters using “limma” and “ggpubr” R packages. Subsequently, we computed the immune score of each patient by the ESTIMATE algorithm which was implemented through R package “ESTIMATE”. We utilized R package “ggpubr” to assess the immune score differences between the two clusters along with the high and low-risk groups [74].

### Gene set variation analysis

GSVA is a non-supervised, non-parametric tool commonly used to estimate changes in the biological processes and pathways activities in samples of expression datasets [75]. The “c2.cp.kegg.v6.2.-symbols” gene sets were used to run these GSVAs (*p* < 0.05), and were retrieved from the MSigDB database. We then used the R package “GSVA” investigated differences across activities of pathways between high- and low-risk groups.

### Exploration of the model in the clinical treatment

R packages pRRophtic and ggplot2 were used to compare the half-maximal inhibitory concentration (IC50) differences of chemotherapeutic drugs between the low- and high-risk groups of patients with ESCC on the basis of the Genomics of Drug Sensitivity in Cancer (GDSC) (www.cancerrxgene.org/).

## Supplementary Information

**Additional file 1: Table S1.** The specific risk score and risk level group for each patient with ESCC.

**Additional file 2: Table S2.** Median survival times for the high- and low- risk groups ESCC patients of survival curves stratified by these clinical characteristics in the training, testing, and entire sets.

**Additional file 3: Figure S1.** (**A**, **D**, **G**) The heat map of 10 prognostic DEOSG expressions in the train (**A**), testing (**D**), and entire sets (**G**), respectively. (**B**,** E**,** H**) The comparison of 10 prognostic DEOSG expressions between high- and low-risk groups in the training (**B**), testing (**E**), and entire sets (**H**), respectively. (**C**, **F**, **I**) The correlations among 10 prognostic DEOSG between high- and low-risk groups in the training (**C**), testing (**F**), and entire sets (**I**), respectively.

**Additional file 4: Table S3.** The results of univariate Cox proportional hazards regression analyses between clinical features and risk score in the training, testing, and entire sets.

**Additional file 5: Table S4.** The results of univariate Cox proportional hazards regression analyses between clinical features and risk score in the training, testing, and entire sets.

**Additional file 6: Figure S2.** (**A**, **F**, **K**) Comparison of the relationship between the clinical characteristics of patients between high- and low-risk groups in the training, testing, and entire sets, respectively. (**B**–**E**) The scatter diagram showed the relationship between gender (**B**), clinical stage (**C**), T stage (**D**), N stage (**E**) and the risk score in the training set. (**G**–**J**) The scatter diagram showed the relationship between gender (**G**), clinical stage (**E**), T stage (**F**), N stage (**J**) and the risk score in the testing set. (**L**–**O**) The scatter diagram showed the relationship between gender (**L**), clinical stage (**M**), T stage (**N**), N stage (**O**) and the risk score in the entire set.

## Data Availability

The entire RNA-seq profile data and the clinical data of ESCC patients in this study come from The Cancer Genome Atlas (TCGA, https://cancergenome.nih.gov/) database, and Gene expression omnibus (GEO, GSE53625, GPL18109, https://www.ncbi.nlm.nih.gov/gds/) database. RNA-seq expression profiles of esophageal normal samples were retrieved from the genotype-tissue expression (GTEx, https://commonfund.nih.gov/gtex) database.

## Abbreviations

ESCC: Esophageal squamous cell carcinoma
DEOSGs: Differentially expressed oxidative stress-related genes
EC: Esophageal cancer
GEO: Gene expression omnibus
TCGA: The cancer genome atlas
GTEx: Genotype-tissue expression
GSEA: Gene Set Enrichment Analysis
KEGG: Kyoto encyclopedia of genes and genomes
GO: Gene ontology
ROC: Receiver operating characteristic
AIC: Akaike's information criterion
TIME: Tumor immune microenvironment
GSVA: Gene set variation analysis
IC50: The half-maximal inhibitory concentration
GDSC: Genomics of Drug Sensitivity in Cancer
OS: Overall survival
HRs: Hazard ratios
CDF: Cumulative distribution function
AUC: Area under the curve
TNM stage: Tumor, node, and metastasis stage
T stage: Tumor stage
N stage: Node stage
M stage: Metastasis stage
