# Supplementary figures and images for: Oxidative stress genes in patients with esophageal squamous cell carcinoma: construction of a novel prognostic signature and characterization of tumor microenvironment infiltration
Source: BMC Bioinformatics. 2022 Sep 30;23:406. doi: 10.1186/s12859-022-04956-9 (PMC9523924; doi:10.1186/s12859-022-04956-9)

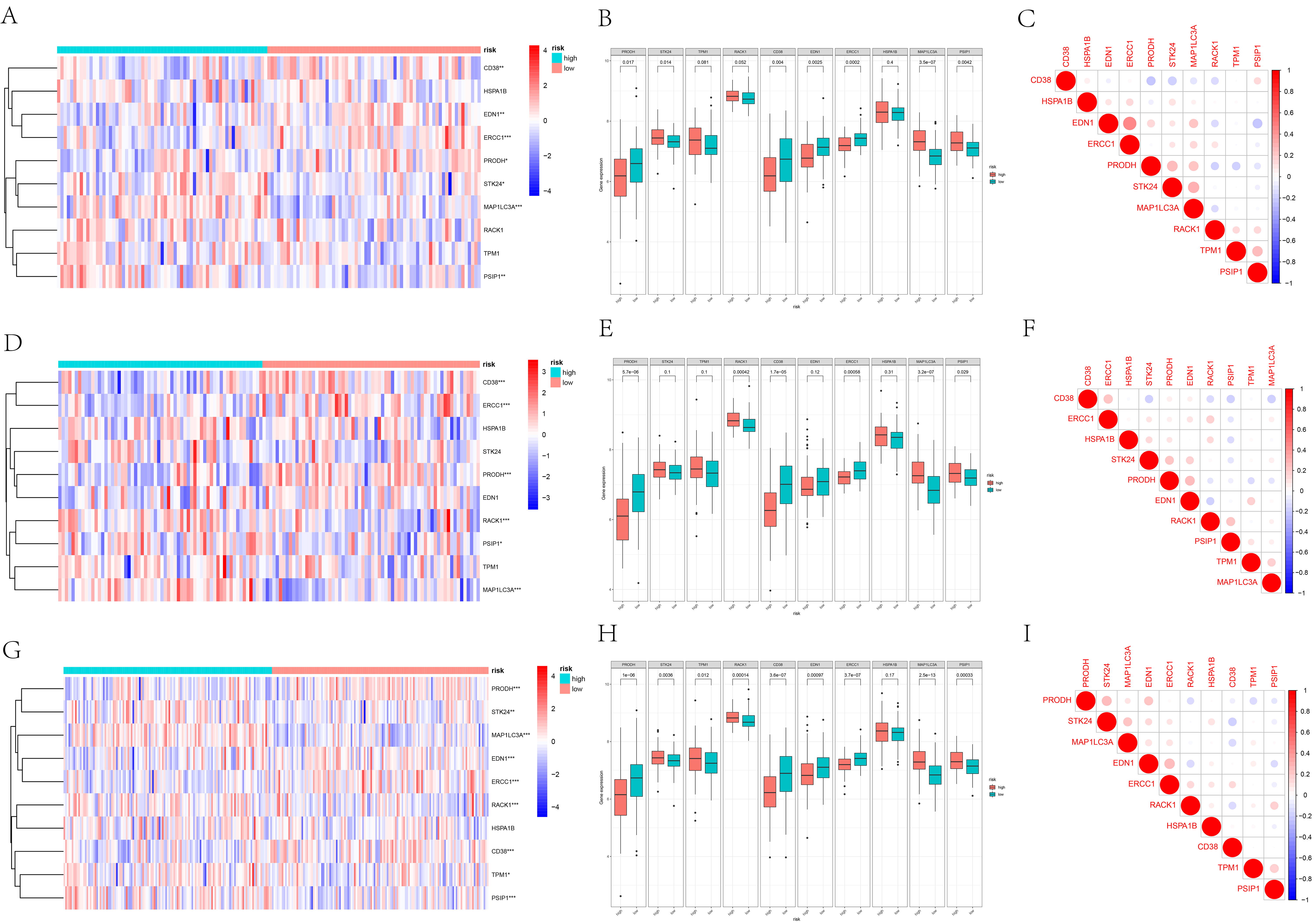

Supplement: Supplementary file 3 — Additional file 3: Figure S1. (A, D, G) The heat map of 10 prognostic DEOSG expressions in the train (A), testing (D), and entire sets (G), respectively. (B, E, H) The comparison of 10 prognostic DEOSG expressions between high- and low-risk groups in the training (B), testing (E), and entire sets (H), respectively. (C, F, I) The correlations among 10 prognostic DEOSG between high- and low-risk groups in the training (C), testing (F), and entire sets (I), respectively. [file 12859_2022_4956_MOESM3_ESM.jpg]

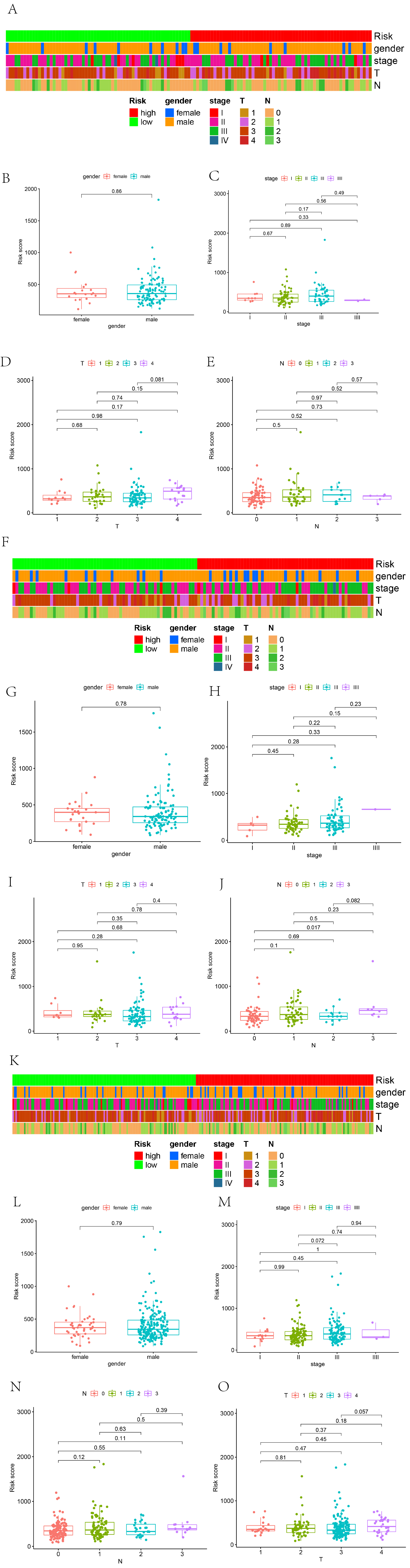

Supplement: Supplementary file 6 — Additional file 6: Figure S2. (A, F, K) Comparison of the relationship between the clinical characteristics of patients between high- and low-risk groups in the training, testing, and entire sets, respectively. (B–E) The scatter diagram showed the relationship between gender (B), clinical stage (C), T stage (D), N stage (E) and the risk score in the training set. (G–J) The scatter diagram showed the relationship between gender (G), clinical stage (E), T stage (F), N stage (J) and the risk score in the testing set. (L–O) The scatter diagram showed the relationship between gender (L), clinical stage (M), T stage (N), N stage (O) and the risk score in the entire set. [file 12859_2022_4956_MOESM6_ESM.jpg]
